# Supplementary material for: Reconciling Mining with the Conservation of Cave Biodiversity: A Quantitative Baseline to Help Establish Conservation Priorities
Source: PLoS One. 2016 Dec 20;11(12):e0168348. doi: 10.1371/journal.pone.0168348 (PMC5173368; doi:10.1371/journal.pone.0168348)
Supplement: S1 Dataset — (ZIP) [file pone.0168348.s002.zip › Taxa/Serra Norte/SN_2013/N4WS/Lista N4WS-09.pdf]

CAVIDADE N4WS-0009

Classe	Ordem	Fam/Outros	Gên/Outros	Espécie	S-U	U-S	Zon.
Arachnida	Acari	(Parasitengonina)		sp.1		X	P
Arachnida	Acari	(Parasitengonina)		sp.2		X	P
Arachnida	Acari	(Parasitengonina)		sp.5		X	P
Arachnida	Acari	Acaridae		jovem		X	P
Arachnida	Acari	Anoetidae		sp.1		X	P
Arachnida	Acari	Cosmochtoniidae		sp.1		X	P/A
Arachnida	Acari	Cunaxidae		sp.1		X	P
Arachnida	Acari	Labidostommatidae		sp.1	X	X	P
Arachnida	Acari	Laelapidae	*Stratiolaelaps*	sp.1		X	P
Arachnida	Acari	Macronyssidae		sp.1		X	P
Arachnida	Acari	Rhagidiidae		sp.1		X	E/P
Arachnida	Acari	Scutacaridae		sp.1		X	P
Arachnida	Amblypygi	Phrynidae	*Heterophrynus*	*longicornis*		1	P
Arachnida	Araneae	Araneidae	*Alpaida*	*septemmammata*	X	X	E/P
Arachnida	Araneae	Drymusidae	*Drymusa*	*spelunca*		X	E
Arachnida	Araneae	Ochyroceratidae	*Speocera*	sp.1	X	X	E/P/A
Arachnida	Araneae	Pholcidae	*Mesabolivar*	*eberhard*	X		A
Arachnida	Araneae	Scytodidae	*Scytodes*	*globula*	5	1	E/P/A
Arachnida	Araneae	Tetragnathidae	*Azilia*	*histrio*	X	X	P/A
Arachnida	Araneae	Theridiidae	*Achaearanea*	sp.1		X	P
Arachnida	Araneae	Theridiosomatidae	*Plato*	sp.1	X	X	E/P
Arachnida	Opiliones	Escadabiidae		sp.2	X	X	P
Arachnida	Opiliones	Sclerosomatidae		jovem		X	P
Arachnida	Pseudoscorpiones	Chernetidae	*Spelaeochernes*	sp.1	X	X	E/P/A
Arachnida	Pseudoscorpiones	Chthoniidae	*Pseudochthonius*	sp.1		X	E
Arachnida	Schizomida	Hubbardiidae	aff. *Rowlandius*	jovem	X	X	P/A
Diplopoda	Polydesmida	Paradoxosomatidae		sp.1		1	P
Diplopoda	Polydesmida	Pyrgodesmidae		sp.1	X	X	P/A
Diplopoda	Polyxenida	Polyxenidae		jovem		X	A
Diplopoda	Siphonophorida	Siphonophoridae		sp.1		1	P
Diplopoda	Spirostreptida	Pseudonannolenidae	*Pseudonannolene*	sp.1		1	P
Entognatha	Collembola	Isotomidae		sp.1		X	E
Entognatha	Collembola	Paronellidae		sp.1	X	X	E/P
Entognatha	Collembola	Symphypleona		sp.1		X	P
Entognatha	Diplura	Campodeidae		sp.1		X	P
Insecta	Blattodea	Blaberidae		jovem		1	P
Insecta	Blattodea	Blattellidae		jovem	1		P
Insecta	Diptera	Cecidomyiidae	Cecidomyiinae	sp.	X	X	E
Insecta	Diptera	Ceratopogonidae		jovem		X	P
Insecta	Diptera	Phoridae	Metopininae	sp.	X		E
Insecta	Diptera	Phoridae	*Trophodeinus*	sp.	X		A
Insecta	Diptera	Psychodidae	*Pericoma*	sp.	X		A
Insecta	Diptera	Psychodidae	*Sciopemyia*	*sordellii*	X	X	E/P/A
Insecta	Diptera	Tipulidae	Tipulinae	sp.	X		E/P
Insecta	Heteroptera	Reduviidae	Emesinae	sp.3	1		P
Insecta	Heteroptera	Reduviidae	Reduviinae	jovem	1	3	E/P
Insecta	Heteroptera	Schizopteridae	Schizopterinae	sp.1		X	E/P
Insecta	Hymenoptera	Braconidae		sp.4		X	P
Insecta	Hymenoptera	Diapriidae		sp.2		X	P

Insecta	Hymenoptera	Formicidae	*Anochetus*	sp.1		X	P
Insecta	Hymenoptera	Formicidae	*Gnamptogenys*	sp.2	X	X	E/P
Insecta	Hymenoptera	Formicidae	*Pachycondyla*	*striata*		1	E
Insecta	Hymenoptera	Formicidae	*Solenopsis*	sp.2		X	E/P
Insecta	Isoptera	Termitidae	*Diversitermes*	sp.		X	P
Insecta	Isoptera	Termitidae	*Nasutitermes*	sp.	X		E/P
Insecta	Lepidoptera	Noctuoidea		sp.1	1	1	P
Insecta	Orthoptera	Phalangopsidae	*Paraclodes*	sp.	1		E
Insecta	Orthoptera	Phalangopsidae	*Phalangopsis*	sp.	30	60	E/P/A
Insecta	Psocoptera	Epipsocidae	*Mesepipsocus*	sp.1		X	P
Insecta	Psocoptera	Psyllipsocidae	*Psyllipsocus*	sp.1		X	P
Insecta	Psocoptera	Psyllipsocidae	*Psyllipsocus*	sp.2	X		P
Malacostraca	Isopoda	Armadillidae		sp.1		X	E/P
Nematomorpha				sp.1	1		P
Mammalia	Chiroptera	Emballonuridae	*Peropteryx*	sp.		1	E
